# Numerical examination of concentration-dependent wastewater sludge ejected into a drinking water source

**DOI:** 10.1038/s41598-023-42026-z

**Published:** 2023-09-20

**Authors:** S. O. Adesanya, T. A. Adeosun, T. A. Yusuf, A. A. Adeyemi, J. S. Bamigboye, A. Ogunlaja, R. S. Lebelo, E. I. Unuabonah

**Affiliations:** 1 Department of Mathematics and Statistics, Redeemer’s University, Ede, Nigeria; 2 Department of Mathematics, Federal College of Education, Iwo, Nigeria; 3 Department of Mathematics, Adeleke University, Ede, Nigeria; 4 Department of Biological Sciences, Redeemer’s University, Ede, Nigeria; 5 Department of Chemical Sciences, Redeemer’s University, Ede, Nigeria; 6 Education Department, Vaal University of Technology, Private Bag X021, Vanderbijlpark, 1911 South Africa; 7 Hydrodynamics Unit, African Center of Excellence for Water and Environmental Research (ACEWATER), Redeemer’s University, Ede, Nigeria

**Keywords:** Environmental sciences, Hydrology, Health care, Risk factors, Mathematics and computing

## Abstract

One of the significant water-related health challenges globally is due to pollutant fate. Contaminants endanger the lives of humans, animals, and even plants. The present mathematical analysis explains reactive wastewater sludge ejected into a drinking water source from wastewater treatment plants. The assumption that wastewater sludge follows a power-law constitutive relation leads to nonlinear momentum and concentration equations. The contaminants are assumed to follow a nonlinear irreversible first-order sorption model. The numerical solution of the coupled problem is solved using the Bivariate Spectral Local Linearization Method and validated with the spectral Chebyshev weighted residual method. Profiles are presented for dimensionless flow velocity and concentration. Comprehensive explanations for the obtained results are provided with relevant applications.

## Introduction

One of nature's greatest gifts to humanity is water, which occupies the most significant area on earth. Besides supporting life, it contributes to the global economy due to its extensive usage in agriculture, industrial, and manufacturing industries. Unfortunately, the massive use of water has led to the discharge of hazardous pollutants into a good fraction of drinking water sources globally. Because of this challenge, many researchers are interested in water treatment to ensure safe drinking water. For instance, Sharma and Bhattacharya^1^ reviewed an array of water contaminants with some purification techniques. They classified contaminants into four categories, i.e., inorganic, organic, biological, and radiological, with appropriate elimination methods. Amrose et al.^2^ reviewed the impact on human health, cost implications of water treatment, and potential risks with some treatment strategies. Gwimbi et al.^3^ reported the adverse effects of biological contaminants in water such as the outbreak of *Escherichia coli* in drinking water sources that caused diarrhea in a Lesotho village. Interested readers can see the work by Lin et al.^4^ for more on pollutant migration and related health hazards.

The dispersive nature of contaminants in water suggests that a tedious analysis based on mathematical modeling can be used to gain insight into the problem. This way, Chinyoka and Makinde^5^ applied the incompressible mass transfer flow technique to explain the nonlinear dispersion of pollutants into a water body. The model formulation considered Newtonian flow with density, diffusion, and viscosity dependence on concentration. Further analysis of the work done in^5^ was carried out recently by Gorder and Vajravelu^6^, from which the exact solution of the steady flow problem was derived together with a numerical solution for the transient case. In^7^, Nwaigwe extended the situation to heat and mass transfer since temperature also affects water quality. Gorder et al.^8^ focused on the self-similar solution of unsteady flow where density and viscosity depend exponentially on concentration. Considering the nonlinear transport properties, Nwaigwe^9^ developed a numerical scheme for the solution of Brinkman flow with variable fluid properties. A two-dimensional analysis for steady water flow through porous walls with concentration-dependent density for flow filtration is presented in^10^.

All the above studies are limited to the Newtonian case in which the effects of wastewater sewage sludge are neglected. Using the wastewater treatment plant as a case study, extremely concentrated sludge with a high percentage of solid contaminant represented a departure from the Newtonian model. Therefore, the solutions obtained in^5–9^ will break down since the shear stress–strain relation cannot be linear in the non-Newtonian fluid. As a result of this behavior, Santos et al.^11^ presented a power law rheological model to model sludge behavior from a water treatment plant. Csizmadia and Till ^12^, in their study of a comparative analysis of the power law by Bingham and Hersschel-Bulkley, presented non-Newtonian models for activated sludge. A similar study by Haldebwang et al.^13^ conducted a curve fitting for three different rheological models for both laminar and turbulent cases of sludge in a pipe flow. More exciting results on the rheological properties of wastewater sludge can be found in works reported by Eshtiaghi et al.^14^ and Wei et al.^15^ and some references cited therein.

Motivated by the studies on the non-Newtonian flow documented in^11–14^, reaction kinetics, and the channel porosity effect^10^, the focus here is to investigate the combined impacts on the work done in^5^. The outcome is expected to be useful in treating wastewater sludge from most treatment plants and other related industrial and manufacturing processes where sludge is constantly ejected into drinking water sources. Understanding these flow properties will provide more information to ensure the United Nations’ drive to meet goal #6—the provision of clean water. A thorough literature survey shows that the study is yet to be reported. Therefore, this study on the non-Newtonian sludge flow using the power law constitutive model is innovative. In the next section, the mathematical formulations for the unsteady fully developed flow with constant vertical penetration are presented for both flow momentum and concentration of pollutants. This way, a set of coupled nonlinear partial differential equations will be presented in Sect. "Mathematical analysis" of this paper with detailed mathematical analysis, while Sect. "Bivariate spectral local linearization method of solution" is devoted to constructing a bivariate spectral local quasi-linearization method for the approximation to the initial-boundary value system of partial differential equations.

## Mathematical analysis

The transient flow problem earlier studied by Chinyoka and Makinde^5^ for nonlinear contaminant ejection is revisited. The buoyancy-induced flow along a vertical channel whose x-axis is in flow direction and the y-axis is taken in a perpendicular direction. The flow assumptions are now as follows:

(i) the fluid is dilatant, reactive, and incompressible,
(ii) fully developed condition implies that $\frac{\partial }{\partial x} = 0$ ,
(iii) the leaky channel walls allow constant fluid penetration by suction/injection,
(iv) at a time $t = 0$ with $\;\mu = \mu_{0}$ and $D = D_{0}$,
(v) at $t > 0,$ the fluid properties become,$\mu = \mu \left( C \right),D = D\left( C \right),S = Q\left( C \right)$
(vi) the nonlinear buoyancy dependence on the concentration of the contaminant is neglected.
(vii) slippage is negligible.

Where $\mu ,\mu_{0}$ represents the dynamic and constant component of water viscosity $C,D,$ represents the dissolved pollutant concentration and diffusion coefficient, $D_{0} ,S$ are the referenced diffusion and nonlinear source term components. Due to possibility of leakages of toxic chemical like flocculants from the water treatment plant the model of Chinyoka and Makinde^5^ is modified to include the effect of suction and injection. In this regard, the unsteady coupled governing equations the flow of waste water can be written as:

1 $$ \left. \begin{gathered} \rho \left( {\frac{{\partial \overline{u}}}{{\partial \overline{t}}} + v_{0} \frac{{\partial \overline{u}}}{{\partial \overline{y}}}} \right) = - \frac{\partial P}{{\partial \overline{x}}} + \frac{\partial }{{\partial \overline{y}}}\left( {\mu \left( C \right)\left| {\frac{{\partial \overline{u}}}{{\partial \overline{y}}}} \right|^{m - 1} \frac{{\partial \overline{u}}}{{\partial \overline{y}}}} \right) + \rho g\beta \left( {C - C_{0} } \right),\;\;\;m > 1 \hfill \\ \;\;\frac{{\partial \overline{C}}}{{\partial \overline{t}}} + \rho \left( {\frac{\Gamma }{1 - \Gamma }} \right)\frac{{\partial \overline{C}_{SP} }}{{\partial \overline{t}}} + v_{0} \frac{{\partial \overline{C}}}{{\partial \overline{y}}} = \frac{\partial }{{\partial \overline{y}}}\left( {D\left( C \right)\frac{{\partial \overline{C}}}{{\partial \overline{y}}}} \right) + S\left( C \right) \hfill \\ \end{gathered} \right\} $$

Additional terms in (1) arise due to departure from the Newtonian to non-Newtonian flow behaviour of sludge when $m \ne 1$. Since sludges are mostly emulsions due to particle aggregation, the irreversible coalescence process is represented by the first-order reaction rate $\frac{{\partial \overline{C}_{SP} }}{{\partial \overline{t}}}$. In Eqs. (1) above, $P,\rho ,\overline{u},v_{0} ,\overline{t},\overline{x},\overline{y},\Gamma ,m$ represent the pressure, sludge density, velocity, constant injection/suction velocity, time, axial, vertical coordinates, pores, and power-law index. $\left( {g,\beta } \right)$ are the gravitational acceleration, and coefficient of expansion. The non-symmetrical nature due to wall leakages suggests the initial, no-slip, and non-moving wall boundary conditions.

2 $$ \left. {u\left( {\overline{y},0} \right) = M\left( {1 - \frac{{\overline{y}^{2} }}{{a^{2} }}} \right),\,\,C\left( {\overline{y},0} \right) = C_{0} ,\;\;\;u\left( { \pm a,\,\overline{t}} \right) = 0,\,\,C\left( { \pm a,\,\overline{t}} \right) = C_{w} ,\,{\text{for }}\,\overline{t} > 0} \right\} $$

$M,a,C_{w} ,C_{0}$ are initial condition control parameters, channel half width, and referenced wall concentrations. Following Serato^16^, the solid reactive contaminant $\overline{C}_{SP}$ in the sludge follows the irreversible first-order sorption model of the form

3 $$ \frac{{\partial C_{SP} }}{\partial t} = K_{1} \left( {C - C_{0} } \right)^{n} $$

The dependence of dynamic viscosity, diffusion, and nonlinear source on pollutant concentration are defined as (kindly refer to Chinyoka and Makinde^5^):

4 $$ \;\;\mu \left( C \right) = \mu_{0} e^{{b_{1} \left( {C - C_{0} } \right)}} ,\,\,D\left( C \right) = D_{0} e^{{b_{2} \left( {C - C_{0} } \right)}} ,\,\,\,S\left( C \right) = Qe^{{b_{3} \left( {C - C_{0} } \right)}} , $$

$\mu_{0} ,D_{0} ,Q,\;b_{{i\left( {i = 1,2,3} \right)}}$ denote the constant component of viscosity and variation coefficients. First-order rate constant, reactive solid contaminant, and external source coefficients respectively.

with the following variables and parameters,

5 $$ \left. \begin{gathered} y = \frac{{\overline{y}}}{a},\,x = \frac{{\overline{x}}}{a},u = \frac{{\overline{u}a}}{\nu },\,t = \frac{{\nu \overline{t}}}{{a^{2} }},\,P = \frac{{\overline{p}a^{2} }}{{\rho \nu^{2} }},K = - \frac{\partial P}{{\partial x}},\upsilon = \frac{{\mu_{0} }}{\rho },\mu = \frac{{\overline{\mu }}}{{\mu_{0} }},Gc = \frac{{g\beta a^{3} \left( {C_{w} - C_{0} } \right)}}{{\upsilon^{2} }},Sc = \frac{\upsilon }{{D_{0} }}, \hfill \\ D = \frac{{\overline{D}}}{{D_{0} }},\left( {\alpha ,\gamma ,\beta } \right) = b_{1,2,3} \left( {C_{w} - C_{0} } \right),\;k_{1} = \frac{{\rho K_{1} a^{2} \Gamma_{d} \left( {C_{w} - C_{0} } \right)^{n - 1} }}{\upsilon },R = \frac{{v_{0} a}}{\upsilon },\;\lambda = \frac{{a^{2} Q}}{{\left( {C_{w} - C_{0} } \right)\upsilon }},S = \left| {\frac{\upsilon }{{a^{2} }}} \right|^{m} \frac{{a^{2} }}{\upsilon } \hfill \\ \end{gathered} \right\} $$

For dilatant fluids with positive values of the index *m*, we get,

6 $$ \left. \begin{gathered} \frac{\partial u}{{\partial t}} + R\frac{\partial u}{{\partial y}} = K + \frac{\partial }{\partial y}\left( {e^{\alpha \phi } \left( {\frac{\partial u}{{\partial y}}} \right)^{m} } \right) + Gc\phi ,\;\;\; \hfill \\ \;\;\frac{\partial \phi }{{\partial t}} + R\frac{\partial \phi }{{\partial y}} = \frac{1}{Sc}\frac{\partial }{\partial y}\left( {e^{\gamma \phi } \frac{\partial \phi }{{\partial y}}} \right) + \lambda e^{\beta \phi } - k_{1} \phi^{n} \hfill \\ \end{gathered} \right\} $$

with initial and boundary conditions,

7 $$ \left. {u\left( {y,0} \right) = 0.1\left( {1 - y^{2} } \right),\,\,\phi \left( {y,0} \right) = 0,\;\;u\left( { \pm 1,\,t} \right) = 0,\,\,\phi \left( { \pm 1,\,t} \right) = 1,\,{\text{for }}\,t > 0} \right\} $$

In (5), $\left( {u,\phi ,m} \right)$, dimensionless velocity and pollutant concentration, power-law index, $\left( {R,K,\alpha ,Gc} \right)$ represents suction Reynolds number, constant pressure gradient, viscosity variation parameter and solutal Grashof number, $\left( {Sc,\gamma ,\lambda } \right),$ Schmidt number, concentration dependent parameter, nonlinear source parameter, $\left( {\beta ,n,k_{1} } \right)$ pollutant variation parameter, order of chemical reaction and coefficient of chemical kinetics.

## Bivariate spectral local linearization method of solution

In the following section, the procedure for the computable form of the system of Eqs. (6), (7) will be presented based on bivariate spectral local linearisation approximation theory. Following Bellman and Kalaba^17^, including^18–20^ we set

8 $$ \left. \begin{gathered} \Omega_{1} = K + \frac{\partial }{\partial y}\left( {e^{\alpha \phi } \left( {\frac{\partial u}{{\partial y}}} \right)^{m} } \right) + Gr\phi - \frac{\partial u}{{\partial t}} - R\frac{\partial u}{{\partial y}},\;\;\; \hfill \\ \Omega_{2} = \;\;\frac{1}{Sc}\frac{\partial }{\partial y}\left( {e^{\gamma \phi } \frac{\partial \phi }{{\partial y}}} \right) + \lambda e^{\beta \phi } - k_{1} \phi^{n} - \frac{\partial \phi }{{\partial t}} - R\frac{\partial \phi }{{\partial y}}. \hfill \\ \end{gathered} \right\} $$

Such that the quasi-linearized version of (6) becomes

9 $$ \left. \begin{gathered} a_{0,r} (y,t)\frac{{\partial^{2} u_{r + 1} }}{{\partial y^{2} }} + a_{1,r} (y,r)\frac{{\partial u_{r + 1} }}{\partial y} + a_{2,r} (r,t)\frac{{\partial u_{r + 1} }}{\partial t} = R_{1,r} (y,t) \hfill \\ b_{0,r} (y,t)\frac{{\partial^{2} \phi_{r + 1} }}{{\partial y^{2} }} + b_{1,r} (y,t)\frac{{\partial \phi_{r + 1} }}{\partial y} + b_{2,r} (y,t)\phi_{r + 1} + b_{3,r} (y,t)\frac{{\partial \phi_{r + 1} }}{\partial t} = R_{2,r} (y,t) \hfill \\ \end{gathered} \right\} $$

where the coefficients used in (9) are defined as

10 $$ \left. \begin{gathered} a_{0,r} (y,t) = \frac{{\partial \Omega_{1} }}{{\partial \left( {u_{yy} } \right)}} = me^{\alpha \phi (y,t)} \left( {\frac{\partial u}{{\partial y}}(y,t)} \right)^{m - 1} ,\,\, \hfill \\ a_{1,r} (y,r) = \frac{{\partial \Omega_{1} }}{{\partial \left( {u_{y} } \right)}} = \alpha me^{\alpha \phi (y,t)} \frac{\partial \phi }{{\partial y}}(y,t)\left( {\frac{\partial u}{{\partial y}}(y,t)} \right)^{m - 1} + (m - 1)m\frac{{\partial^{2} u}}{{\partial y^{2} }}(y,t)e^{\alpha \phi (y,t)} \left( {\frac{\partial u}{{\partial y}}(y,t)} \right)^{m - 2} - R, \hfill \\ a_{2,r} (r,t) = \frac{{\partial \Omega_{1} }}{{\partial \left( {u_{t} } \right)}} = - 1,\,\,b_{0,r} (r,t) = \frac{{\partial \Omega_{2} }}{{\partial \left( {\phi_{yy} } \right)}} = \frac{{{\text{e}}^{\gamma \phi (y,t)} }}{Sc},\,b_{1,r} = \frac{{\partial \Omega_{2} }}{{\partial \left( {\phi_{y} } \right)}} = \frac{{2\gamma e^{\gamma \phi (y,t)} }}{{{\text{Sc}}}}\frac{\partial \phi }{{\partial y}}(y,t) - R, \hfill \\ b_{2,r} = \frac{{\partial \Omega_{2} }}{\partial \left( \phi \right)} = k_{1} ( - n)\phi (y,t)^{n - 1} + \lambda ne^{\beta \phi (y,t)} + \frac{{\gamma e^{\gamma \phi (y)} }}{{{\text{Sc}}}}\left( {\gamma \left( {\frac{\partial \phi }{{\partial y}}(y,t)} \right)^{2} + \frac{{\partial^{2} \phi }}{{\partial y^{2} }}(y,t)} \right),\,b_{3,r} = \frac{{\partial \Omega_{2} }}{{\partial \left( {\phi_{t} } \right)}} = - 1 \hfill \\ R_{1,r} = \left( {a_{0,r} (y,t)\frac{{\partial^{2} u_{r} }}{{\partial y^{2} }} + a_{1,r} (y,t)\frac{{\partial u_{r} }}{\partial y} + a_{2,r} (y,t)\frac{{\partial u_{r} }}{\partial t}} \right) - \Omega_{1,r} (y,t), \hfill \\ R_{2,r} = \left( {b_{0,r} (y,t)\frac{{\partial^{2} \phi_{r} }}{{\partial y^{2} }} + b_{1,r} (y,t)\frac{{\partial \phi_{r} }}{\partial y} + b_{2,r} \phi_{r} + b_{3,r} \frac{{\partial \phi_{r} }}{\partial t}} \right) - \Omega_{2,r} (y,t). \hfill \\ \end{gathered} \right\} $$

It is well known that set of polynomials is dense in the set of continuous functions, therefore, to obtain the solution of the (6) subject to (7), we seek a series solution that is based on Lagrange cardinal polynomial, $L_{p} \left( y \right)L_{q} \left( \tau \right)$, approximation of the form:

11 $$ \left. \begin{gathered} u(y,t) \approx U(y,t) = \sum\limits_{p = 0}^{{N_{y} }} {\sum\limits_{q = 0}^{{N_{\tau } }} {U(y_{p} ,\tau_{q} )} } L_{p} \left( y \right)L_{q} \left( \tau \right), \hfill \\ \phi (y,t) \approx \Phi (y,t) = \sum\limits_{p = 0}^{{N_{y} }} {\sum\limits_{q = 0}^{{N_{\tau } }} {\Phi (y_{p} ,\tau_{q} )} } L_{p} \left( y \right)L_{q} \left( \tau \right), \hfill \\ \end{gathered} \right\} $$

with the bivariate Chebyshev Gauss–Lobatto points

12 $$ y_{i} = \left\{ {cos(\frac{\pi i}{{N_{y} }})} \right\}_{i = 0}^{{N_{y} }} ,\tau_{j} = \left\{ {cos(\frac{\pi j}{{N_{\tau } }})} \right\}_{j = 0}^{{N_{\tau } }} $$

In which time,$\,t \in [0,\,T^{\prime}]\,$ is transform into Chebyshev domain $\tau \in [ - 1,1]$ using $t = \frac{T^{\prime}(\tau + 1)}{2}$ and

13 $$ L_{p} \left( y \right) = \prod\limits_{{\begin{array}{*{20}c} {i = 0} \\ {i \ne k} \\ \end{array} }}^{{N_{x} }} {\frac{{y - y_{k} }}{{y_{i} - y_{k} }}} ,\;\;\;L_{q} \left( \tau \right) = \prod\limits_{{\begin{array}{*{20}c} {j = 0} \\ {j \ne k} \\ \end{array} }}^{{N_{\tau } }} {\frac{{\tau - \tau_{k} }}{{\tau_{i} - \tau_{k} }}} ,\quad L_{p} (y_{k} ) = \delta_{ik} = \left\{ {\begin{array}{*{20}l} {0,i \ne k} \hfill \\ { \, 1,i = k} \hfill \\ \end{array} } \right. $$

At every Chebyshev-Gaus-Lobatto points,$(y_{i} ,\tau_{j} )$, derivatives appearing in (9) can be computed easily based on the following Chebyshev differentiation matrix procedure:

14 $$ \begin{gathered} \frac{{\partial^{r} U}}{{\partial y^{r} }}(y_{i} ,\tau_{j} ) = \sum\limits_{p = 0}^{{N_{x} }} {D_{i,p}^{r} U(y_{p} ,\tau_{j} )} = {\mathbf{D}}^{r} {\mathbf{U}}_{j} , \hfill \\ \frac{\partial U}{{\partial \tau }}(y_{i} ,\tau_{j} ) = \sum\limits_{q = 0}^{{N_{\tau } }} {d_{j,q} U(y_{i} ,\tau_{q} ) = \sum\limits_{q = 0}^{{N_{\tau } }} {d_{j,q} {\mathbf{U}}_{q} } } , \hfill \\ \frac{{\partial^{r} \Phi }}{{\partial y^{r} }}(y_{i} ,\tau_{j} ) = \sum\limits_{p = 0}^{{N_{x} }} {D_{i,p}^{r} \Phi (y_{p} ,\tau_{j} )} = {\mathbf{D}}^{r} {{\varvec{\Phi}}}_{j} , \hfill \\ \frac{\partial \Phi }{{\partial \tau }}(y_{i} ,\tau_{j} ) = \sum\limits_{q = 0}^{{N_{\tau } }} {d_{j,q} \Phi (y_{i} ,\tau_{q} )} = \sum\limits_{q = 0}^{{N_{\tau } }} {d_{j,q} {{\varvec{\Phi}}}_{q} } \hfill \\ \end{gathered} $$

In (14), ${\mathbf{D}}\,and\,{\mathbf{d}}_{j,q} = \frac{T}{2}d_{j,q}$ represent the Chebyshev differentiation matrices for $(N_{y} + 1) \times (N_{y} + 1)$ and $(N_{\tau } + 1) \times (N_{\tau } + 1)$ respectively while ${\mathbf{U}}_{j}$ and ${{\varvec{\Phi}}}_{j}$ are given by:

15 $$ \begin{gathered} {\mathbf{U}}_{j} = [U(y_{0} ,\tau_{j} ),U(y_{1} ,\tau_{j} ),U(y_{2} ,\tau_{j} ),...,U(y_{{N_{y} }} ,\tau_{j} )]^{T} ,\,for\,j = 0,1,2,...,N_{\tau } , \hfill \\ {{\varvec{\Phi}}}_{j} = [\Phi (y_{0} ,\tau_{j} ),\Phi (y_{1} ,\tau_{j} ),\Phi (y_{2} ,\tau_{j} ),...,\Phi (y_{{N_{y} }} ,\tau_{j} )]^{T} ,for\,j = 0,1,2,...,N_{\tau } , \hfill \\ \end{gathered} $$

with superscript T and *I* denoting the transpose and identity matrices, respectively. Substituting (15) and (14) into (9) we get

16 $$ \begin{gathered} a_{0,r} ({\mathbf{y}},\tau_{j} ){\mathbf{D}}^{2} {\mathbf{U}}_{r + 1,j} + a_{1,r} ({\mathbf{y}},\tau_{j} ){\mathbf{DU}}_{r + 1,j} + a_{{_{2,r} }} ({\mathbf{y}},\tau_{j} )\sum\limits_{q = 0}^{{N_{\tau } }} {{\mathbf{d}}_{j,q} {\mathbf{U}}_{r + 1,q} } = R_{1,r} ({\mathbf{y}},\tau_{j} ), \hfill \\ b_{{_{0,r} }} ({\mathbf{y}},\tau_{j} ){\mathbf{D}}^{{\mathbf{2}}} {{\varvec{\Phi}}}_{r + 1,j} + b_{1,r} ({\mathbf{y}},\tau_{j} ){\mathbf{D\Phi }}_{r + 1,j} + b_{2,r} ({\mathbf{y}},\tau_{j} )I + b_{3,r} ({\mathbf{y}},\tau_{j} )\sum\limits_{q = 0}^{{N_{\tau } }} {{\mathbf{d}}_{j,q} {{\varvec{\Phi}}}_{r + 1,q} } = R_{2,r} ({\mathbf{y}},\tau_{j} ), \hfill \\ \end{gathered} $$

The transformed boundary conditions are:

17 $$ \left. \begin{gathered} U_{r + 1} \left( {y_{0} ,t_{j} } \right) = 0,\;\;\phi_{r + 1} \left( {y_{0} ,t_{j} } \right) = 1, \hfill \\ U_{r + 1} \left( {y_{{N_{y} }} ,t_{j} } \right) = 0,\;\;\phi_{r + 1} \left( {y_{{N_{y} }} ,t_{j} } \right) = 1.\;\; \hfill \\ \end{gathered} \right\} $$

The vector ${\mathbf{U}}_{{r + 1,N_{\tau } }}$ and ${{\varvec{\Phi}}}_{{r + 1,N_{\tau } }}$ correspond to the initial condition given in Eq. (7). Matrices (15) are solved iteratively until suitable results are obtained. The accuracy of the computation is validated by using the regular bivariate Chebyshev collocation method and the results are presented as Tables 1 and 2 in the results and discussions section.

**Table 1:** Validation of Bivariate spectral local linearization scheme for flow velocity when $R=1, m=1.001, Gr=0.1, \alpha = 0.1, Sc=0.6, \gamma = 0.1, \lambda = 0.5, \beta = 0.1, = 0.1, n=2$.

y	$u_{BSLLM} \left( {t,y} \right)$	$u_{SCCM} \left( {t,y} \right)$	$\left| {u_{BSLLM} - u_{SCCM} } \right|$
− 1.00	$-6.0408\times {10}^{-15}$	$0.$	$6.0408\times {10}^{-15}$
− 0.75	$0.160733711995193$	$0.16073505946276145$	1.34747 $\times {10}^{-6}$
− 0.50	$0.292873897785165$	$0.29287717318671447$	3.2754 $\times {10}^{-6}$
− 0.25	$0.390544924564238$	$0.3905509613238606$	6.03676 $\times {10}^{-6}$
0.00	$0.446250046309746$	$0.44626066732462566$	1.0621 $\times {10}^{-5}$
0.25	$0.450174114493185$	$0.45018729934505364$	1.31849 $\times {10}^{-5}$
0.50	$0.38940268585581$	$0.38941193332921603$	9.24747 $\times {10}^{-6}$
0.75	$0.246908398046832$	$0.24691326752861403$	4.86948 $\times {10}^{-6}$
1.00	$3.2174\times {10}^{-14}$	$4.7091\times {10}^{-24}$	3.21739 $\times {10}^{-14}$

**Table 2:** Validation of Bivariate spectral local linearization scheme for dissolved concentration when $R=1, m=1.001, Gr=0.1, \alpha = 0.1, Sc=0.6, \gamma = 0.1, \lambda = 0.5, \beta = 0.1, = 0.1, n=2$.

Y	$\phi_{BSLLM}$	$\phi_{SCCM}$	$\left| {\phi_{BSLLM} - \phi_{SCCM} } \right|$
− 1.00	$0.999999999999587$	$1.$	4.13003 $\times {10}^{-13}$
− 0.75	$1.041339747059161$	$1.0413396661520284$	8.09071 $\times {10}^{-8}$
− 0.50	$1.072276736376537$	$1.0722767363762256$	3.11529 $\times {10}^{-13}$
− 0.25	$1.093843773942933$	$1.0938438366967427$	6.27538 $\times {10}^{-8}$
0.00	$1.104144567881861$	$1.1041445678806283$	1.23279 $\times {10}^{-12}$
0.25	$1.101905050526921$	$1.1019050521119447$	1.58502 $\times {10}^{-9}$
0.50	$1.08541501125928$	$1.0854150112598626$	5.82645 $\times {10}^{-13}$
0.75	$1.053016711600263$	$1.053016625105047$	8.64952 $\times {10}^{-8}$
1.00	$1.000000000002131$	$1.$	2.13096 $\times {10}^{-12}$

## Results and discussion

In this section, numerical results of the dimensionless problems (6) subject to the initial and boundary conditions (7) are presented graphically and in tabular form to describe the effects of various flow parameters on the concentration and flow fields. The numerical results are computed using the quasi-linearized approach based on the bivariate Lagrange polynomials as the trial function and validated with the spectral Chebyshev collocation method. At this point, the quasi-linear bivariate Langrage method converges faster than SCCM, and the CPU time is more minor even with the power law index greater than unity. In this regard, we have taken a near unity value for the comparative study of the two numerical schemes. As shown in Tables 1 and 2, there is a high level of agreement between the two results. Therefore, the numerical solutions are unique to problems (6) and (7).

**Figure 1:**
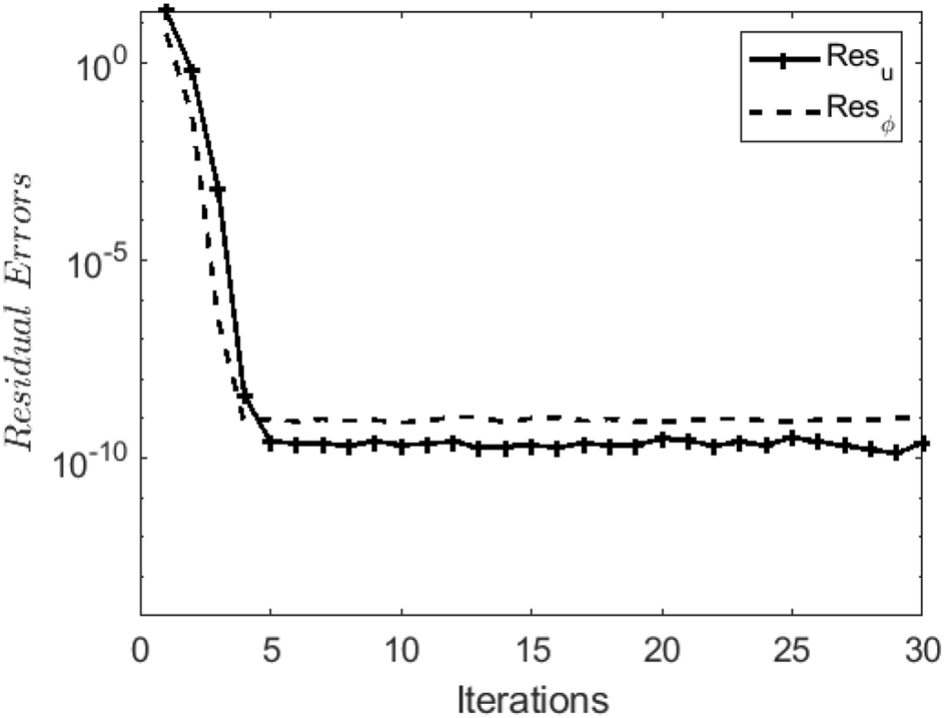
Residual errors.

**Figure 2:**
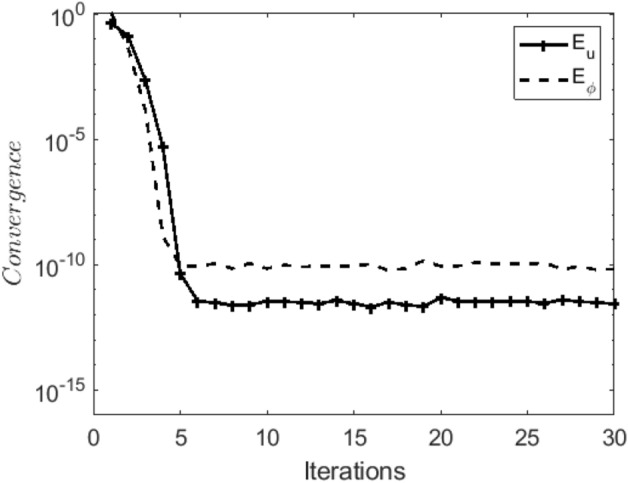
Convergence of residuals.

**Figure 3:**
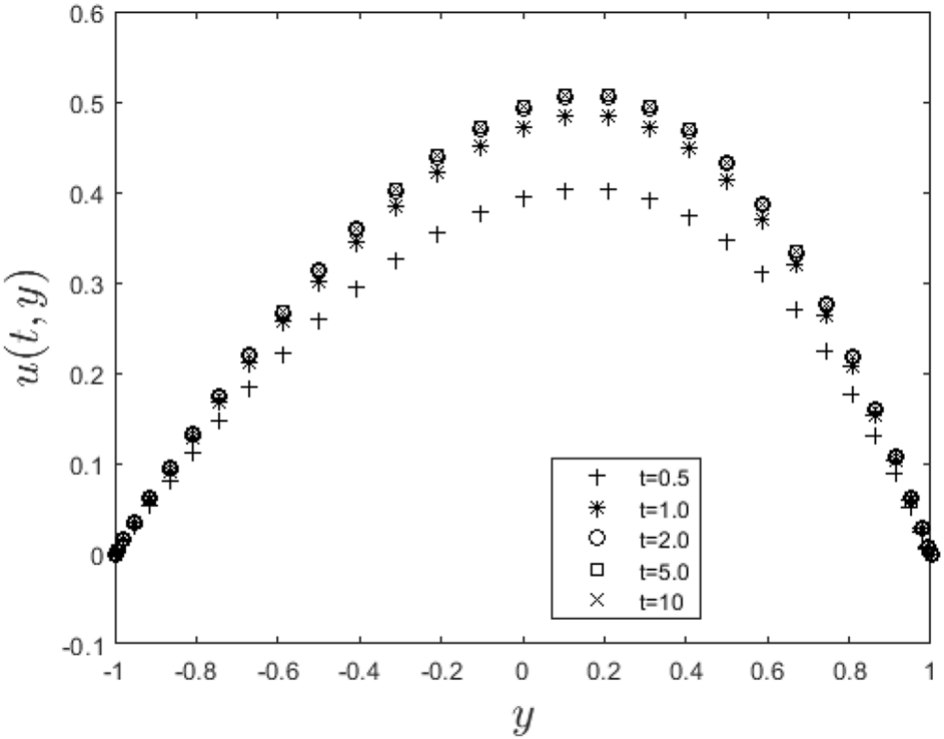
Steady state solution for $u\left( {t,y} \right)$.

**Figure 4:**
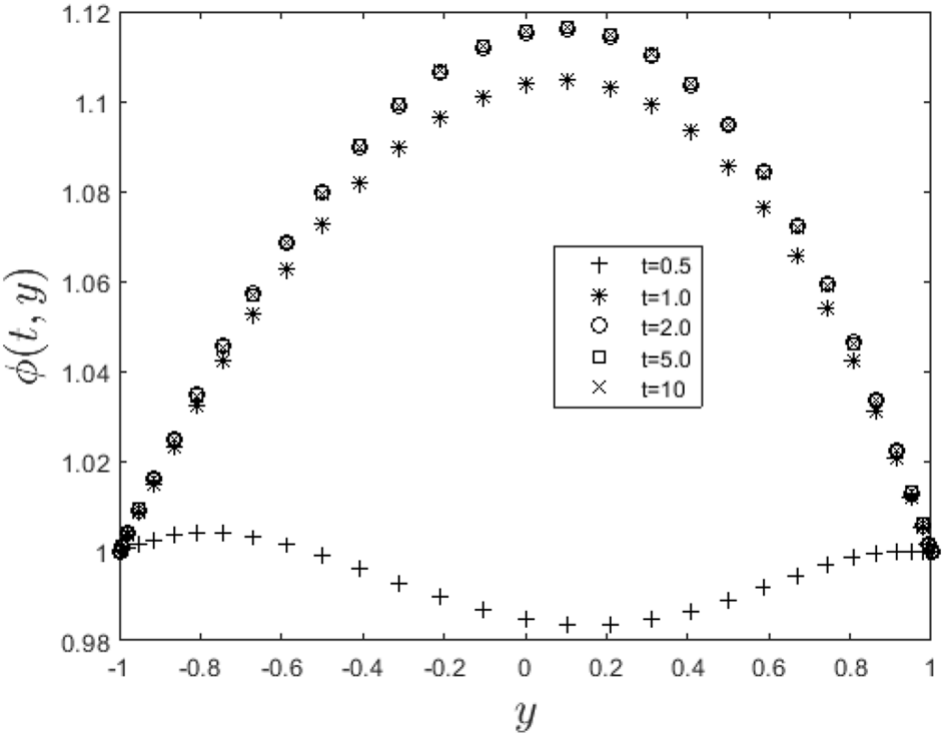
Steady state solution for $\phi \left( {t,y} \right)$.

**Figure 5:**
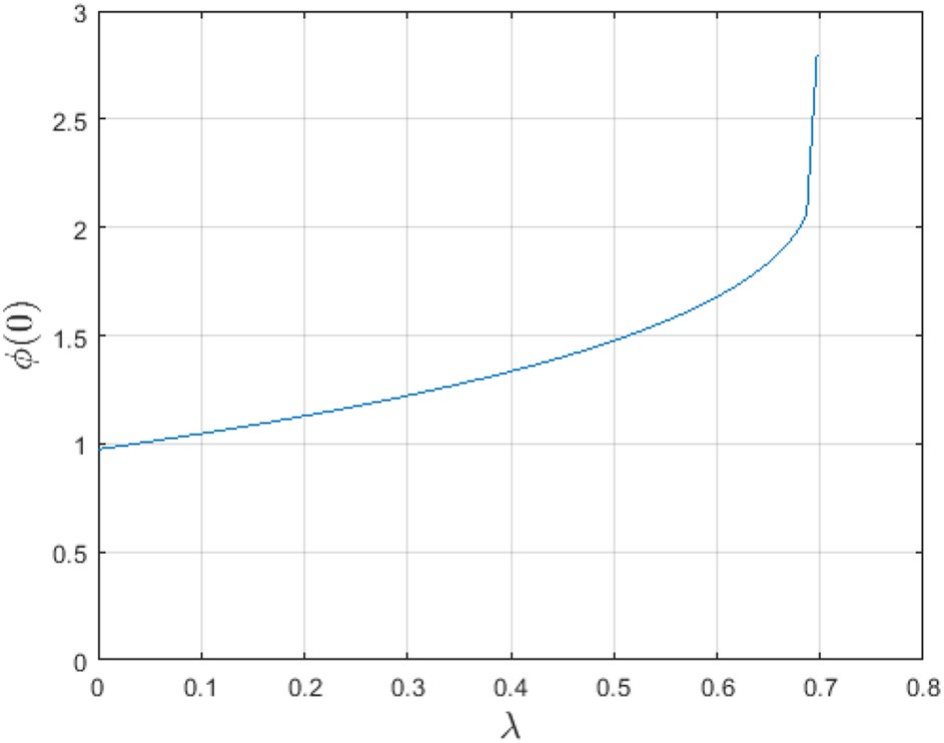
Blow-up solution for $\phi \left( {t,y} \right)$.

**Figure 6:**
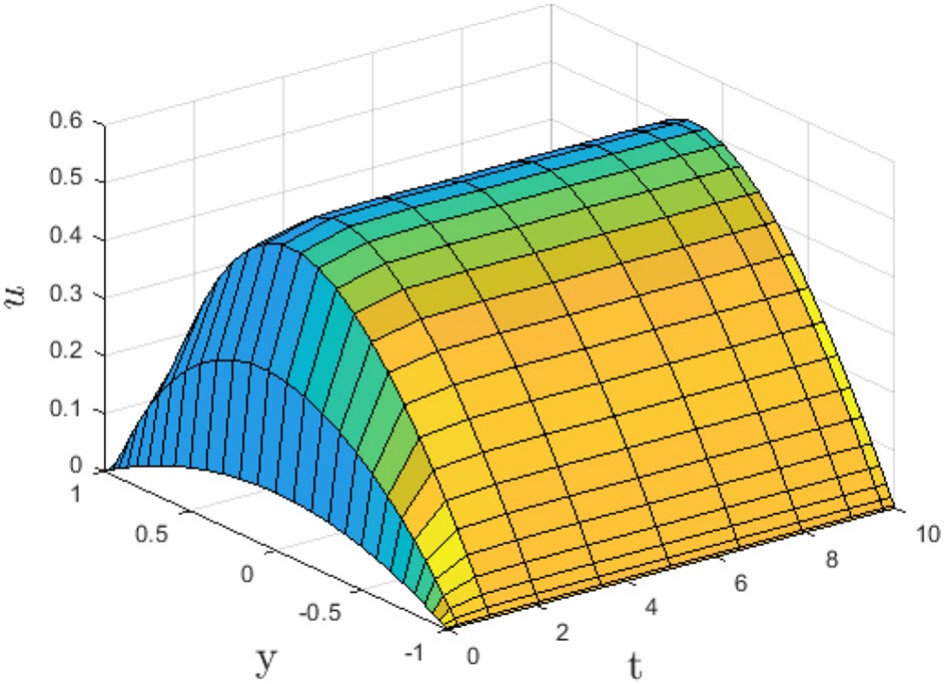
Solution for $u\left( {t,y} \right)$ in 3-D.

**Figure 7:**
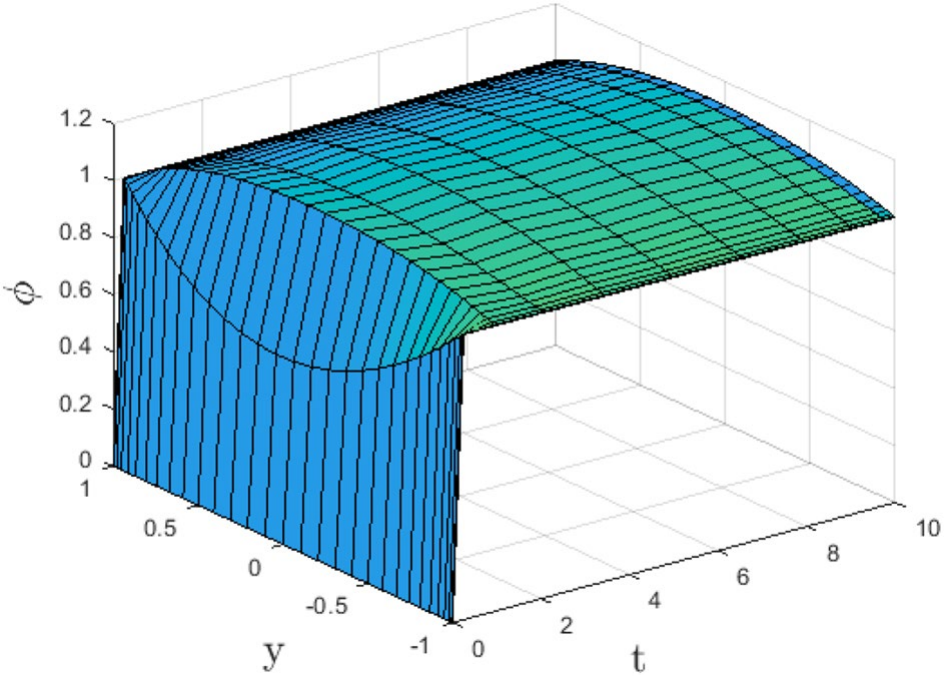
Solution for $\phi \left( {t,y} \right)$ in 3-D.

**Figure 8:**
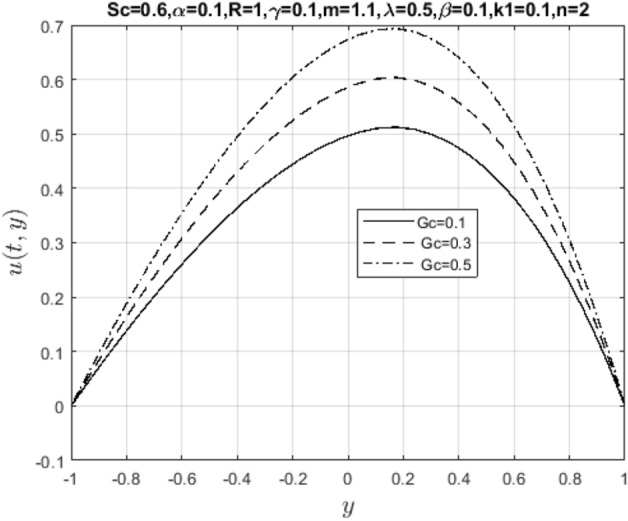
Impact of solutal Grashof number on $u\left( {t,y} \right)$.

**Figure 9:**
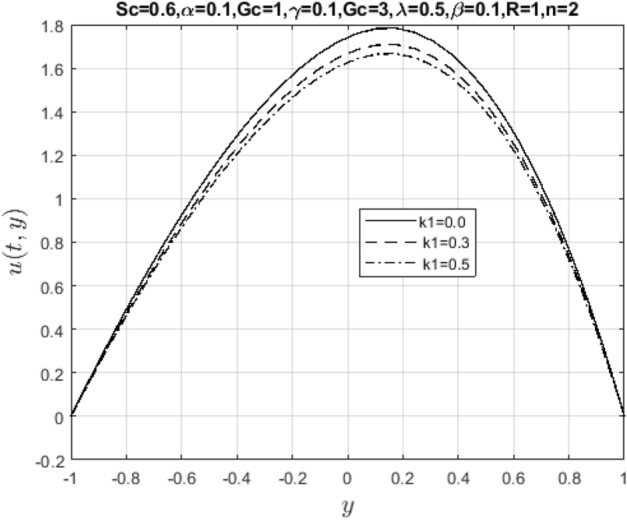
Impact of nonlinear sorption kinetic parameter on $u\left( {t,y} \right)$.

**Figure 10:**
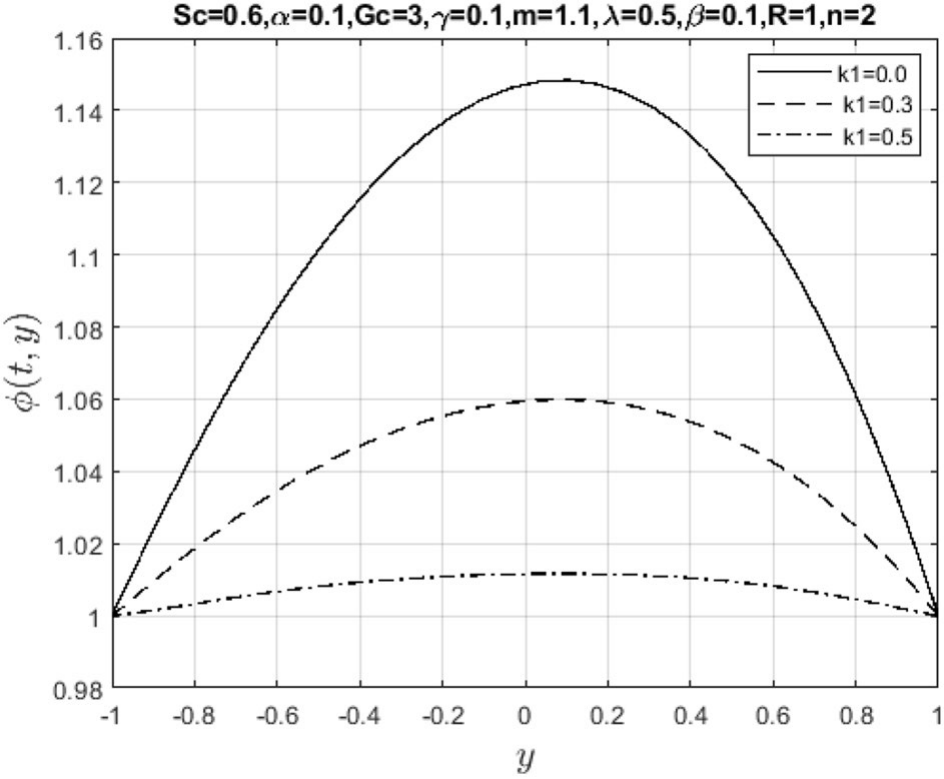
Impact of nonlinear sorption kinetic parameter on $\phi \left( {t,y} \right)$.

**Figure 11:**
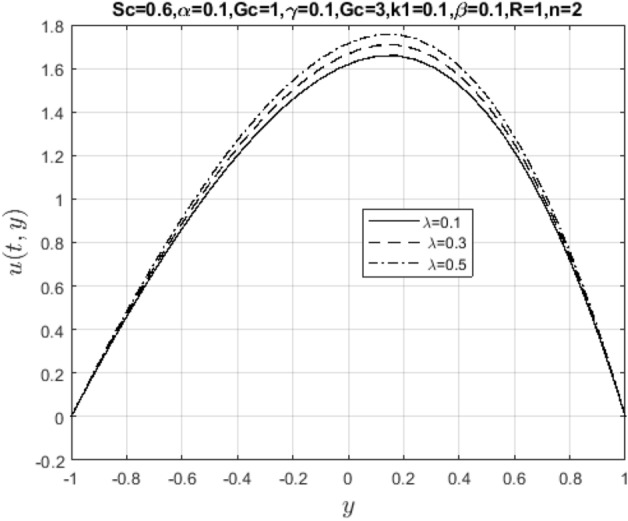
Impact of nonlinear source parameter on $u\left( {t,y} \right)$.

**Figure 12:**
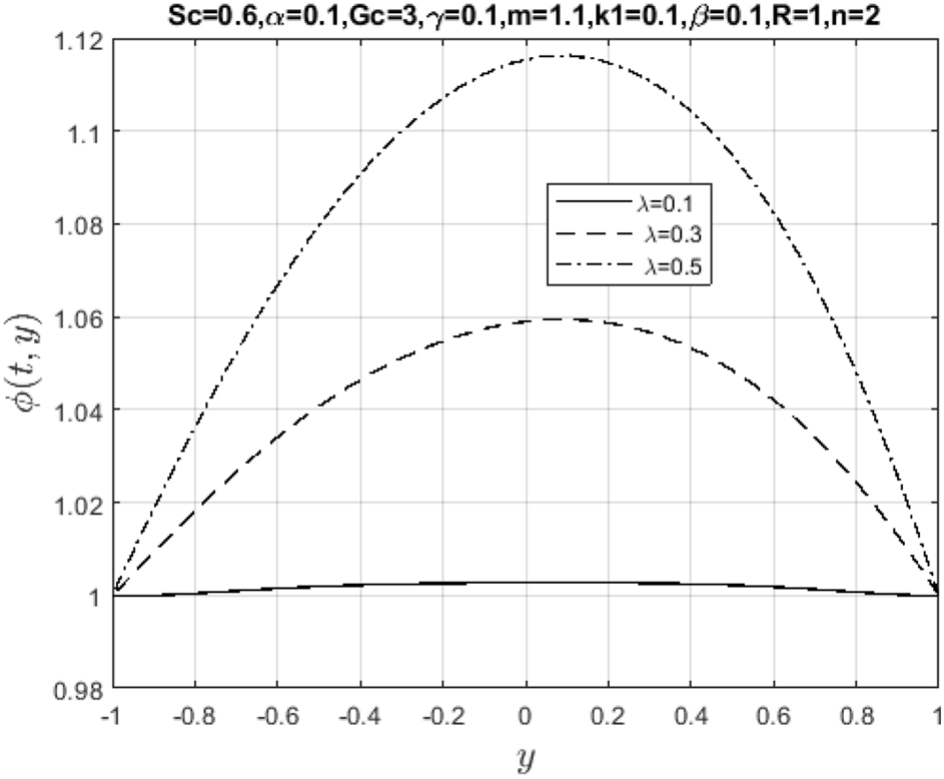
Impact of nonlinear source parameter on $\phi \left( {t,y} \right)$.

In the obtaining Figs. 1, 2, 3, 4, 5, 6 and 7, we have used the following parameter values $R=1, m=1.1, Gr=0.1, \alpha = 0.1, Sc=0.6, \gamma = 0.1, \lambda = 0.5, \beta = 0.1, = 0.1, n=2$. Figure 1 represents the residual error plots when the numerical approximations are returned as solutions to the problem (6), (7). The residual errors are of order 10^–9^ and 10^–10^ for the dimensionless momentum and pollutant concentration, respectively. As a result, the numerical results give excellent residuals of $R_{1,r} \left( {y,t} \right)$ and $R_{2,r} \left( {y,t} \right)$ in (9) and the residual errors converges to 10^–10^ and 10^–11^ respectively as shown in Fig. 2. Figure 3 reveals the steady state behavior of the numerical solutions. Evidently, the solution approached steady state from $t=2$. However, as $t=5$ and above, further solutions are superimposed on $t=2$. Moreover, as seen in Fig. 4, the time-independent solution for pollutant concentration is recovered from $t=2$ while superimpositions are seen afterward beyond this point. Figure 5 reveals the non-global nature of the solution that is typical of all nonlinear PDEs with exponential nonlinearity. The solution is seen to blow up with the nonlinear source term parameter. The 3-D solutions of the initial boundary value problem (6)-(7) are presented in Figs. 6 and 7. Evidently, the solutions are well-behaved as the initial and boundary conditions are well satisfied. Figure 8 shows the impact of buoyance on the flow velocity. As seen from the plot, the flow velocity peak increases with increasing solutal Grashof number values. This results from increasing buoyancy force over viscous force around the core area of the flow channel. Figure 9 depicts the influence of nonlinear sorption kinetic on the flow velocity. The result shows that the wastewater velocity is at its peak without sorption. Further increase in the nonlinear sorption coefficient decreases the flow velocity due to adsorption at the porous walls. In Fig. 10, pollutant concentration is observed to be at maximum when k1, the nonlinear sorption coefficient is absent from the pollutant concentration. Further increase in the parameter resulted in decreased concentration due to the filtration process at the leaking walls. The influence of the nonlinear source term on the flow velocity is presented in Fig. 11. The result shows that the exponential dependence on pollutant concentration enhances flow velocity. This is physically correct due to the convective buoyancy effect on the flow velocity. However, a reverse trend is seen in the spatial variation of pollutant concentration with nonlinear source parameters, as seen in Fig. 12.

**Figure 13:**
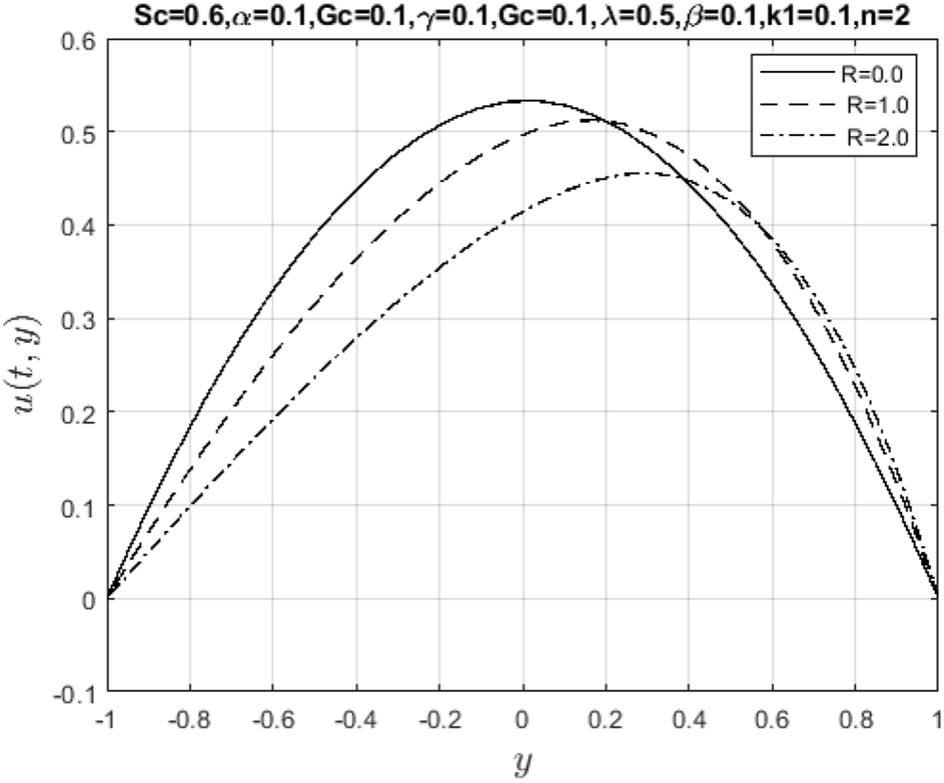
Impact of suction Reynold’s number parameter on $u\left( {t,y} \right)$.

**Figure 14:**
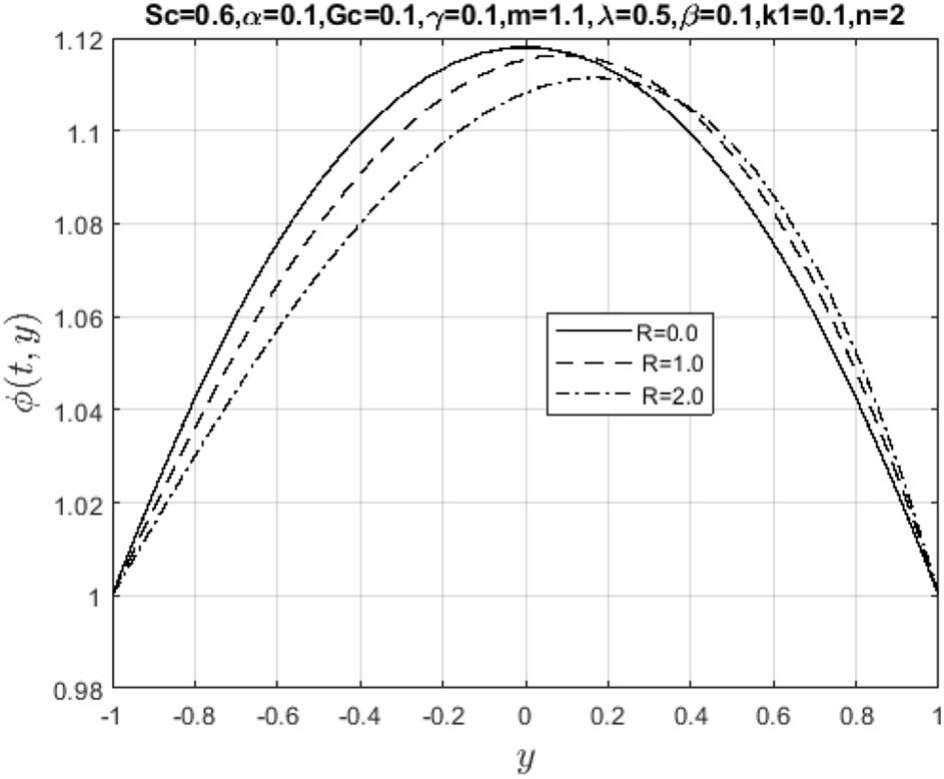
Impact of suction Reynold’s number on $\phi \left( {t,y} \right)$.

**Figure 15:**
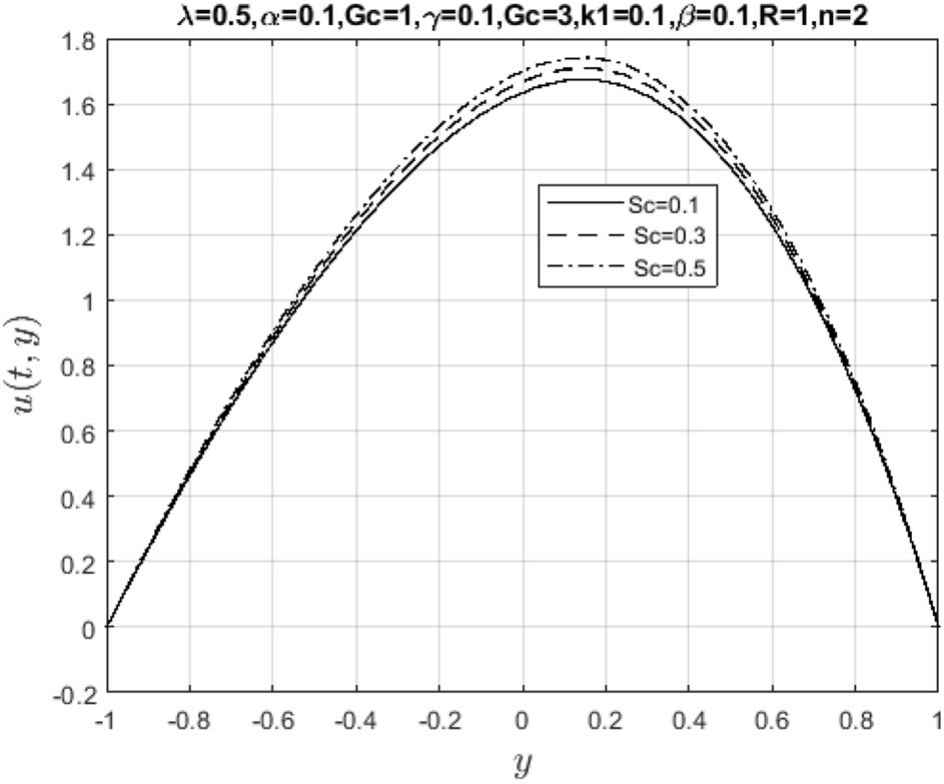
Impact of Schmidt number on $u\left( {t,y} \right)$.

**Figure 16:**
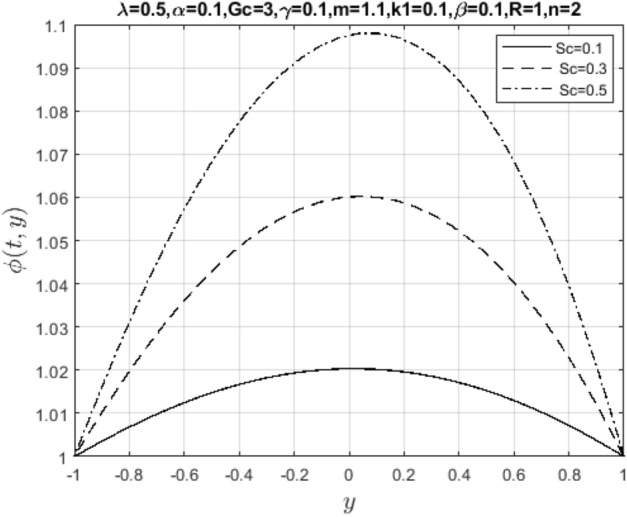
Impact of Schmidt number on $\phi \left( {t,y} \right)$.

Meanwhile, Figs. 13 and 14 represent suction/injection Reynold’s number due to wall porosity. These plots show that the flow retains symmetry if the walls are impervious. Otherwise, the symmetry is lost as the pore opens and becomes wider, and more solid particles in the wastewater are introduced into the channel. Therefore, the flow pattern is distorted through vertical penetration. In bed filtration, Reynold’s number is the main application being used since polluted water is injected into the fixed bed where solid particles are first removed when the pores are more prominent, then filtered water is made to pass through several layers with varying porosity until clean water is collected at the suction end. Lastly, Figs. 15 and 16 show the effect of mass diffusivity and molecular diffusion on the flow and concentration profile. From the plot, pollutant diffusion within the flow channel decreases as the Schmidt number increases. As a result, the concentration of pollutants rises around the deposited point within the channel. Consequently, mass diffusivity increases, and the fluid behaves like a viscoelastic fluid with increasing velocity.

## Conclusion

In this work, a numerical investigation into the unsteady flow of wastewater sludges into drinking water through a porous channel has been carried out. The pollutant is assumed to be chemically reactive and follows the irreversible nonlinear first-order sorption kinetics. The nonlinear governing partial differential equations are formulated based on the fluid dynamics approach and turned into dimensionless problems. The set of nonlinear initial-boundary value problems is solved by the bivariate quasi-linearized model and validated with the spectral Chebyshev collocation method. The significant contributions to knowledge from the present computations are as follows: effect of suction/injection Reynold’s number in flow control is more substantial and cannot be neglected. Irreversible chemical kinetics is significant in the understanding and treatment of wastewater. The influence of solid particles is more pronounced in non-Newtonian flow behaviour wastewater than in Newtonian flow.

In our subsequent formulation, the gravity-driven flow with bioremediation and biochemical oxygen demand effects in contaminated river water flows will be considered under the Monod reaction.

## Data Availability

All data generated or analysed during this study are included in this published article.
